# Confocal Spectroscopy to Study Dimerization, Oligomerization and Aggregation of Proteins: A Practical Guide

**DOI:** 10.3390/ijms17050655

**Published:** 2016-04-30

**Authors:** Yann Gambin, Mark Polinkovsky, Bill Francois, Nichole Giles, Akshay Bhumkar, Emma Sierecki

**Affiliations:** EMBL Australia Node in Single Molecule Sciences, School of Medical Science, the University of New South Wales, Sydney, NSW 2052, Australia; polinkov@gmail.com (M.P.); bfrancoi@clipper.ens.fr (B.F.); n.giles@unsw.edu.au (N.G.); a.bhumkar@unsw.edu.au (A.B.)

**Keywords:** single molecule spectroscopy, number and brightness analysis, protein folding, protein oligomerization, protein-protein interactions

## Abstract

Protein self-association is a key feature that can modulate the physiological role of proteins or lead to deleterious effects when uncontrolled. Protein oligomerization is a simple way to modify the activity of a protein, as the modulation of binding interfaces allows for self-activation or inhibition, or variation in the selectivity of binding partners. As such, dimerization and higher order oligomerization is a common feature in signaling proteins, for example, and more than 70% of enzymes have the potential to self-associate. On the other hand, protein aggregation can overcome the regulatory mechanisms of the cell and can have disastrous physiological effects. This is the case in a number of neurodegenerative diseases, where proteins, due to mutation or dysregulation later in life, start polymerizing and often fibrillate, leading to the creation of protein inclusion bodies in cells. Dimerization, well-defined oligomerization and random aggregation are often difficult to differentiate and characterize experimentally. Single molecule “counting” methods are particularly well suited to the study of self-oligomerization as they allow observation and quantification of behaviors in heterogeneous conditions. However, the extreme dilution of samples often causes weak complexes to dissociate, and rare events can be overlooked. Here, we discuss a straightforward alternative where the principles of single molecule detection are used at higher protein concentrations to quantify oligomers and aggregates in a background of monomers. We propose a practical guide for the use of confocal spectroscopy to quantify protein oligomerization status and also discuss about its use in monitoring changes in protein aggregation in drug screening assays.

## 1. Introduction

### 1.1. Oligomerization: Function and Dysfunction

**Figure 1:**
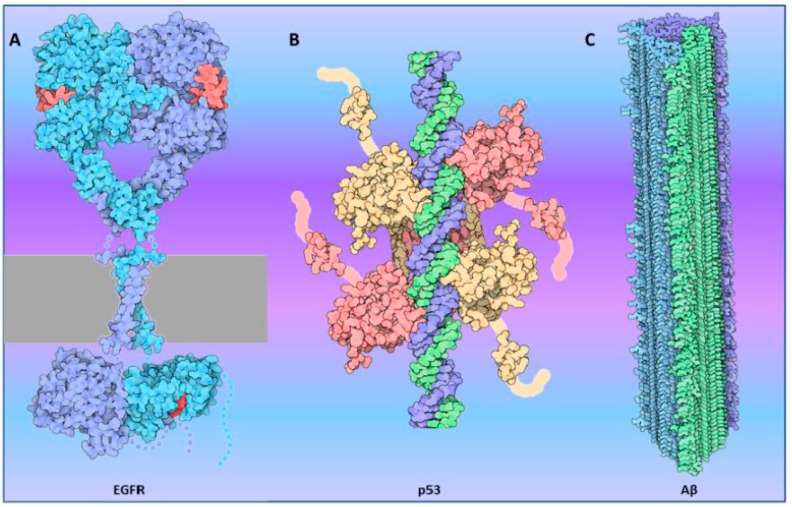
Examples of oligmerization in active proteins: (**A**) Epidermal Growth Factor Receptor (EGFR, shown in light blue and purple) dimerizes in the plasma membrane (represented in gray). Binding of the hormone (in red) triggers activation of the intracellular kinase domain (constructed from PDB entry 2gs6); (**B**) p53 binds to the DNA (blue/green) as a tetramer (shown in pink/yellow) (constructed from PDB entries 1tup , 1olg and 1ycq) ; (**C**) Aβ (1–42) forms fibrils that can accumulate in the brain causing neurodegenerative disorders including Alzheimer’s disease. Three different β-sheet stacks (colored in green, blue and purple) form the amyloid fibril (constructed from PDB entry 2m4j). These examples were “molecules of the month” on the RCSB PDB, generated by David S. Goodsell [17].

Most types of proteins can modulate their function and binding partners by modifying their oligomerization status [1,2]. One of the most described functions of dimerization/oligomerization is the activation/inhibition of enzymatic activities, particularly in kinases. The role of dimerization in signaling has been extensively studied in the case of the receptor tyrosine kinases (RTKs) [3,4,5,6]. A remarkable amount of work has led to a precise description of molecular mechanisms of Epidermal Growth Factor Receptor (EGFR) activation upon ligand binding (Figure 1A), and the role of the dimer [7,8,9,10,11]. This knowledge provided new therapeutic avenues in the treatment of cancers where RTKs are often mutated or over-expressed [12,13,14,15]. Dimerization of receptors also provides a way to activate signaling kinases in non-RTK receptors. One such example is the dimerization of Janus Kinase 2 (JAK2) and the sliding mechanism that leads to its activation following the Growth Hormone Receptor (GHR) binding to its ligand [16].

Oligomerization can also switch the function of a given protein. Remarkably, transcription factors use dimerization and oligomerization as a way to not only increase their affinity for DNA [18,19,20,21,22] (Figure 1B) but also to modulate the subset of genes to be regulated [23,24,25,26] or vary the binding partners that can be recruited [27,28]. For example, for transcription factors involved in circadian oscillations, variation in their oligomeric status is a simple and efficient way to modulate activity [29]. Finally, oligomers of a transcriptional activator can be repressors [30] and *vice versa* [31].

Larger scale oligomerization or polymerization can also find a role in biology. Some functions in the cell, such as endocytosis, vesicle transport [32,33] or cytoskeletal organization [34], are so complex that it requires a full machinery to be carried out. Proteins involved in such mechanisms have the ability to self-assemble to create nanoscale objects of various shape and function. Polymerization is also a process that allows for fast, non-linear signal amplification. This is particularly relevant in immunity where one danger signal must be enough to activate a full-scale response by the organism. Recently, two adaptor proteins of the immune system, the Mitochondrial Antiviral Signaling (MAVS) protein [35,36] and the apoptosis-associated speck-like protein containing a CARD (ASC) [37] have been shown to polymerize upon activation. These proteins actually adopt a prion-like behavior in order to amplify the danger signals not only intracellularly but also in neighboring cells [38]. These discoveries are reigniting the interest in prion-like behavior as a physiologically relevant and beneficial process in mammalian cells [39,40,41].

However, thus far, most examples of protein aggregation, polymerization and prion-like behavior occur in the context of diseases [42,43,44]. The best characterized examples of deleterious protein aggregation and prion-like behavior are found in neurodegenerative diseases [45,46,47,48]. In particular, misfolding and aggregation of proteins, such as Aβ [49] (Figure 1C), α-synuclein [50,51] and Tau [52,53], relevant to Alzheimer’s and Parkinson’s diseases, have been studied at length. More recently, prion-like behavior has been linked to cancer [54,55] as mutations in p53 [56,57] notably lead to polymerization of the protein.

Whether it is a controlled, defined process or an exponential progression, a physiological response to a stimulus or an acquired pathological mutation, oligomerization is a fundamental property of most proteins. Information on the proteins’ oligomerization state is therefore essential to the molecular understanding of many biological events. Though many technologies [58,59,60,61,62,63,64] have been developed to study oligomerization and polymerization, single molecule counting and confocal spectroscopy techniques [65,66,67,68,69] are particularly well suited to the study of protein self-assembly.

### 1.2. Single Molecule Detection and Confocal Spectroscopy for the Study of Protein Self-Assembly

Fluorescence confocal spectroscopy relies on the detection of fluorescent proteins freely diffusing in and out of the confocal volume of a microscope. Confocal spectroscopy methods are especially attractive as they avoid “ensemble” averaging of the properties of the sample. As a small number of proteins are measured at the same time, sub-populations can be detected in complex mixtures. Two methods, Fluorescence Correlation Spectroscopy (FCS) and single-molecule spectroscopy, rely on confocal microscopy to observe the diffusion of fluorescently labeled proteins and track their oligomerization. Both are commonly used to study proteins behavior but have specific limitations.

FCS [70,71,72,73] is often referred to as a “small ensemble” technique as multiple proteins are detected simultaneously in the focal volume (typically at nanomolar concentrations). The method relies on observing of intensity fluctuations as fluorescent particles diffuse into and out of the confocal detection volume. In FCS, a correlator is used to calculate the temporal auto-correlations of the fluorescent signal. This method is extremely powerful as it can yield information on many timescales, from microsecond dynamics (rapid intramolecular fluctuations can be measured by measuring fast changes in the photophysics or environment on the fluorophores) to milliseconds (to measure diffusion parameters). This precise measure of diffusion is especially useful when a small diffusing object (small molecule, peptide or small protein) can bind to a larger particle, creating a large shift in the diffusion coefficient. However, FCS is not optimal when the sample is heterogeneous. The brightest events overwhelm the correlation and dominate the analysis as bursts of very large amplitude create long-range temporal correlations.

“Pure” single-molecule spectroscopy [74,75,76,77,78,79] is performed at picomolar (pM) concentrations, allowing the characterization of highly heterogeneous mixtures. Single molecule spectroscopy is used by many groups to measure Förster Resonance Energy Transfer (FRET) at the single molecule level, but it can also be used to measure the size of small oligomers. The measurements detect individual bursts of fluorescence when a single protein or single oligomer diffuses within the detection volume. Although proteins transiting through the focal volume produce signals with different intensities, depending on their individual trajectories, sufficient sampling of many trajectories can be provide information on the number of proteins in each oligomer [80,81,82,83]. To simplify, the maximal number of photons collected in a burst of a millisecond can be compared with the number of photons emitted by a monomeric fluorophore, and interpreted in terms of the number of proteins in an oligomer [84]. These “pure” single molecule counting techniques are powerful but have limited ability to handle the presence of rare and large objects as the extreme dilution required by single-molecule counting makes rare events virtually undetectable.

The principles of single molecule detection can be applied in a straightforward manner to detect large oligomers at higher protein concentrations, by detecting larger bursts of fluorescence in a background of monomers. Photon counting [85,86] or Number and Brightness (N&B) [87,88,89,90,91,92] analysis, a method often used in laser scanning microscopy in cells, can be viewed as a hybrid approach that can overcome the limitations of FCS and “pure” single molecule spectroscopy. The simple analysis of the heterogeneity of intensity values measured in short time traces yields an extremely robust and precise measure of protein oligomerization. Performed at “FCS” concentrations (nM), the brightness analysis allows the detection of rare events, yet the counting system attributes the same weight to a monomer or a large oligomer, providing a precise picture of the mixture composition. Here, we describe a simple application of the N&B analysis to identify the size of oligomers and demonstrate the use of the brightness parameter (B) as a screening tool for protein aggregation.

## 2. Characterization of the Brightness Parameter in Confocal Spectroscopy

### 2.1. Theory

**Figure 2:**
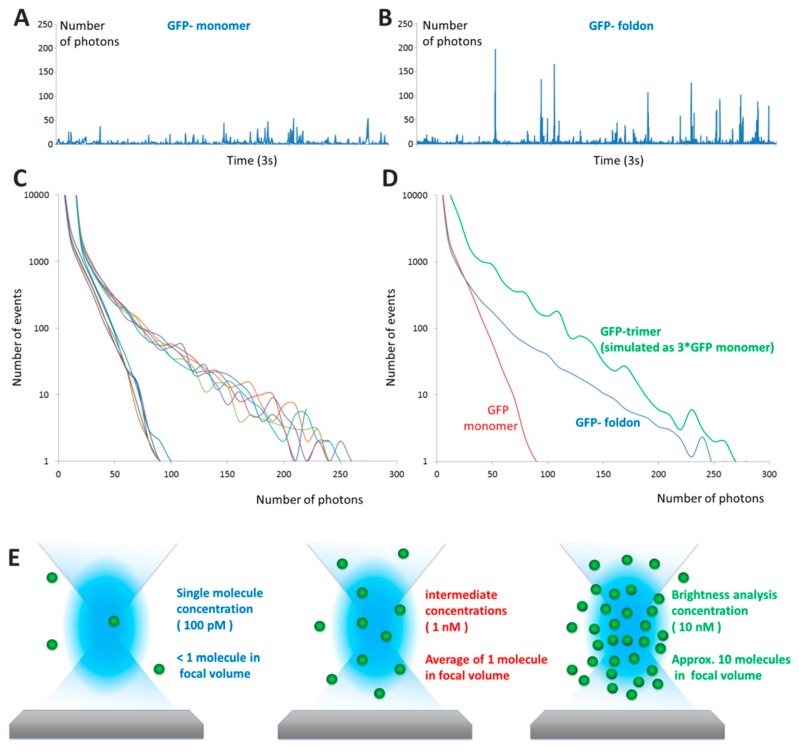

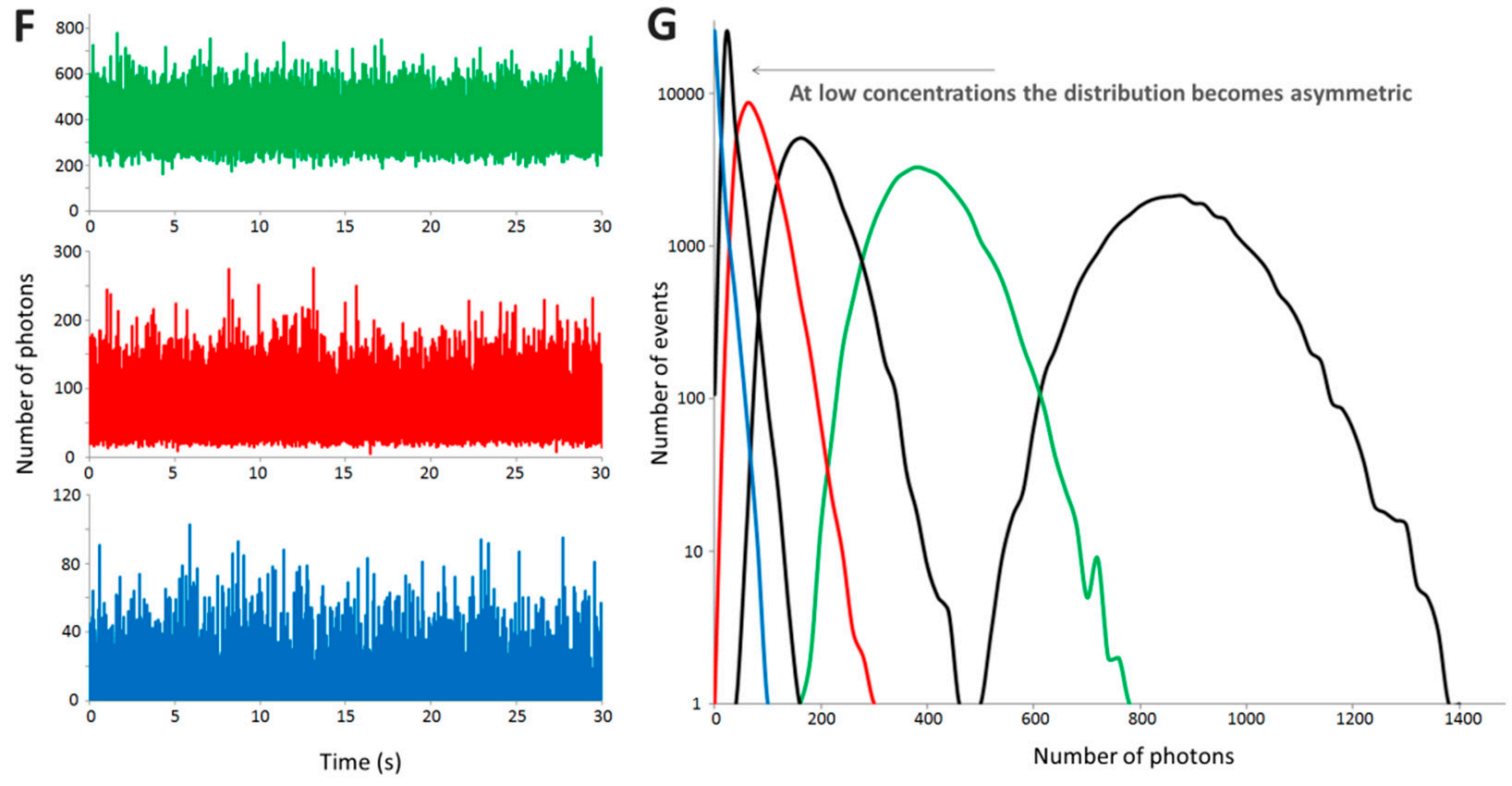
From photon counting histograms to brightness analysis: (**A**,**B**) Typical time traces of fluorescence obtained in “pure” single-molecule experiments for GFP (**A**) and GFP-foldon (foldon is an artificial trimerization domain) (**B**) The number of photons detected is often referred to as “counts per millisecond” (cpms); (**C**) Photon Counting Histograms obtained for 5 distinct experiments for each protein, the colors indicate the different repeats. The GFP curves converge to a maximal value of 80 photons per ms whereas the GFP-foldon proteins reaches a maximum of 250 photons per ms; (**D**) A simulated GFP trimer photon counting histogram obtained by multiplying a monomer trace by three and analyzed for the distribution of values; the data show that the slope of the distribution matches with that of the experiments; (**E**) As concentration of the sample increases from 100 pM to 10 nM, the average number of molecules in the confocal volume increases from <1 to 10 molecules/ms. A typical focal volume is 200–300 nanometers in width and 2–6 times larger in the vertical axis; (**F**) The increase of the average number of molecules is reflected by the increase in background of a typical fluorescent time traces obtained for different concentrations of GFP; (**G**) The distribution of number of events changes from asymmetric at low concentrations to a Gaussian profile. The colors correspond to the time traces in (**F**); intermediate concentrations are shown in black.

For simplicity, let us consider purely monomeric proteins first. At single molecule concentrations, typically picomolar (pM), proteins transiting through the focal volume will produce signal of different brightness, depending on their individual trajectories (Figure 2A,B). This allows for the single molecule brightness analysis illustrated in Figure 2C,D. When the protein concentration is raised significantly (by a factor of ~50–100), the brightness distribution becomes Gaussian as shown in Figure 2G.

**Figure 3:**
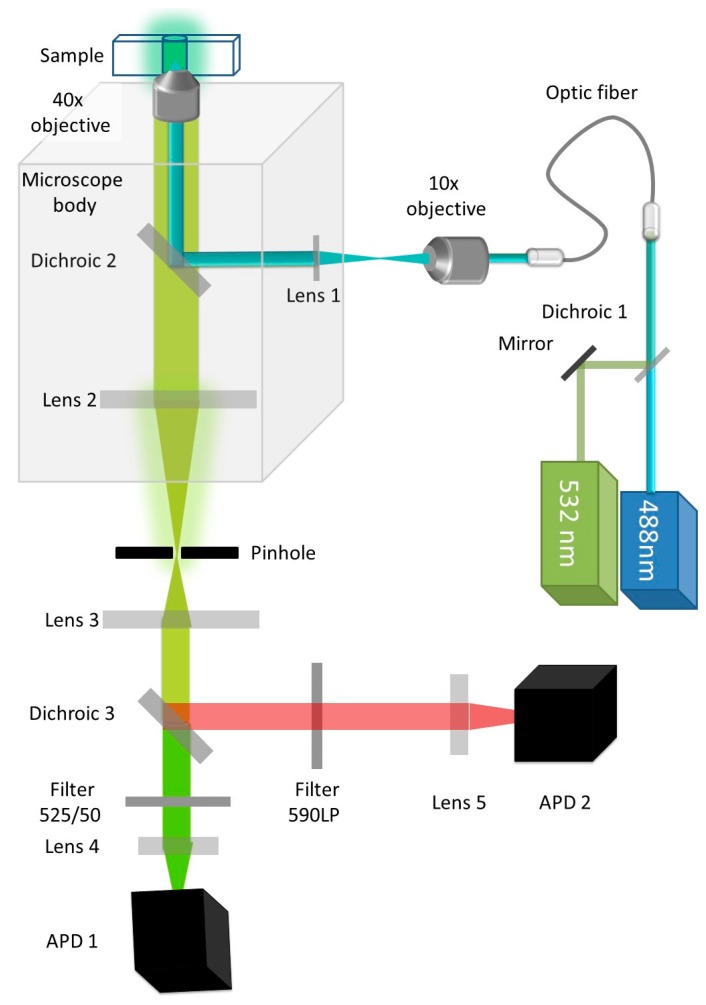
Schematic of the single molecule spectroscopy setup: In the brightness analysis mode, the 532 nm laser is turned off but this setup can also be used for single molecule coincidence or single molecule FRET. A 488 nm laser is used to illuminate the sample containing GFP. The light is focused and the resulting fluorescence is collected through a 40× magnification, 1.2 Numerical Aperture (NA) water immersion objective. In the dual color mode, the fluorescence from GFP and Cherry is separated by a dichroic filter. Fluorescence from GFP is filtered by a 505–540 band pass filter. The number of photons collected in 1 ms time bins is recorded as IGFP (t). Avalanche Photo Diodes (APDs) are used for photon counting.

The signal measured from each fluorescently labeled particle as it diffuses through the focal volume depends on a multitude of factors. These include the path the particle takes, the geometry of the focal volume, and the characteristics of the excitation laser beam, among others. The shape of the confocal volume is extremely important to obtain correct fitting of FCS measurements. Acquisition of FCS data typically requires extensive optimization of all optical elements and perfect alignment of a pinhole with small aperture (typically 30–50 microns) (see Figure 3). On the contrary, brightness measurements are more forgiving as a monomeric reference sample can be used to calibrate each apparatus. The contribution of all these geometrical parameters is integrated in the characteristic width of the histogram for a sample’s time series.

For symmetrical distributions, the variance depends on the lateral and vertical extension of the confocal volume and the protein’s concentration. Notably, the protein’s concentration imparts a Poisson dependence on the distribution, meaning that for a concentration *n*, the distribution has the following characteristics:

(1) $$\begin{array}{c} \mu_{n}=\frac{n}{n_{ref}}\times \;\mu_{ref}\\ \sigma_{n}^{2}=\frac{n}{n_{ref}}\times \sigma_{ref}^{2} \end{array}\}$$

where, $\;\mu_{n}\;$and $\sigma_{n}^{2}$ are the mean and variance for the distribution at a concentration n, while $\mu_{ref}\;$and $\sigma_{ref}^{2}$ are the mean and variance for the distribution at a reference concentration ($n_{ref}$).

The brightness parameter (B) is defined as the ratio of the variance $\sigma^{2}\;$over the mean intensity μ (mathematically, the index of dispersion).

(2) $$B=\frac{\sigma^{2}}{\mu }$$

*B* incorporates all the contributions from the physical characteristics of the system, as described earlier. Mathematically, it follows that *B* should remain invariant with concentration. Indeed, this property serves to confirm the suitability of the model (see Section 2.3 below).

(3) $$B=\frac{\sigma_{n}^{2}}{\mu_{n}}=\frac{\sigma_{ref}^{2}}{\mu_{ref}}$$

Because an oligomer is a complex of monomers, its signal is a multiple of that of the monomer if we assume that the fluorophores are randomly oriented in the oligomer. In turn, it follows that for a given mean signal, *μ*, if the sample consists of dimers, its variance will be double that of a monomer distribution. Consider the distribution of dimers with concentration *n/2* (the concentration of fluorophores is *n*). It follows that$\;\mu_{dimer}=2\;\times \;\mu_{n/2}=\mu_{n}$ [93].

However, the particles pass through the focal volume in pairs, meaning that their distributions are exactly correlated [93].

(4) σdimer2=σn/22+σn/22+2 Cov[f(n2),f(n2)]σdimer2=2 σn/22+2 σn/22σdimer2=2 σn2

By extension, for an N-mer, with fluorophore concentration of *n*,

(5) $$\begin{array}{c} \mu_{olig}=\mu_{n}\\ \sigma_{olig}^{2}=N\times \sigma_{n}^{2} \end{array}\}$$

Again, as $B=\frac{\sigma^{2}}{\mu }$, in the case of an oligomer:$\;B_{olig}=\frac{\sigma_{olig}^{2}}{\mu_{olig}}=\frac{N\times \sigma_{n}^{2}}{\mu_{n}}=N\times B_{mono}$.

Once *B* is known for a pure monomer, it becomes possible to ascertain how other proteins deviate from this behavior by measuring *B* for their time series. Therefore, the size of the oligomer (*N*) can be simply deduced provided that a reference monomeric sample has been characterized using the same setup.

### 2.2. Experimental Measure of the Size of Oligomers

Obtaining a reference monomeric sample is readily accomplished when one uses genetically encoded fluorophores, as their fluorescence properties are independent of the protein they label. Because most laboratories use GFP and its variants for cell microscopy, most proteins are available as GFP fusions for expression in mammalian cells. The brightness can be analyzed directly after cell lysis and centrifugation to remove nuclei and membrane debris. Alternatively, cell-free expression systems such as the eukaryotic system used in our laboratory [94] can be used to produce the fluorescently tagged proteins. This approach shortcuts labeling, denaturation/refolding and purification steps that could affect the protein oligomerization status.

#### 2.2.1. Experimental Setup

The experimental setup we use is described in Figure 3. Single molecule spectroscopy was performed as described previously [84,95]. The measurement requires 20 μL of diluted sample that is placed into a custom-made 192-well silicone plate with a 70 × 80 mm glass coverslip (ProSciTech, Kirwan, QLD, Australia). Plates are analyzed at room temperature on a Zeiss Axio Observer microscope with a custom-built data acquisition setup. Illumination is provided by a 488 nm laser beam, focused in the sample volume using a 40× magnification, 1.2 Numerical Aperture water immersion objective (Zeiss, Oberkochen, Germany). The fluorescence of GFP is measured through a 525/20 nm band pass filter, and detected by a photon counting detector (Micro Photon Devices, Bolzano, Italy). Photons are collected in 1 ms time bins.

#### 2.2.2. Examples: Monomers, Dimers and Trimers

In the first example, eGFP and eGFP-Foldon were expressed in our cell-free system [94] and analyzed. Foldon is a trimeric β-hairpin propeller derived from the C-terminal domain of the T4 fibritin; this domain drives the trimeric assembly of the fibritin and has been used as an efficient artificial trimerization domain [96].

**Figure 4:**
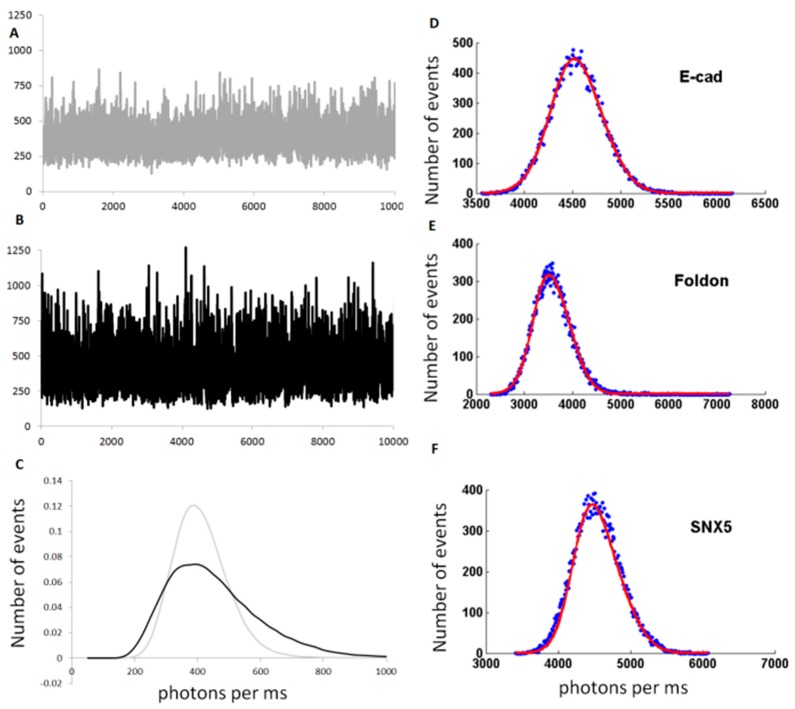

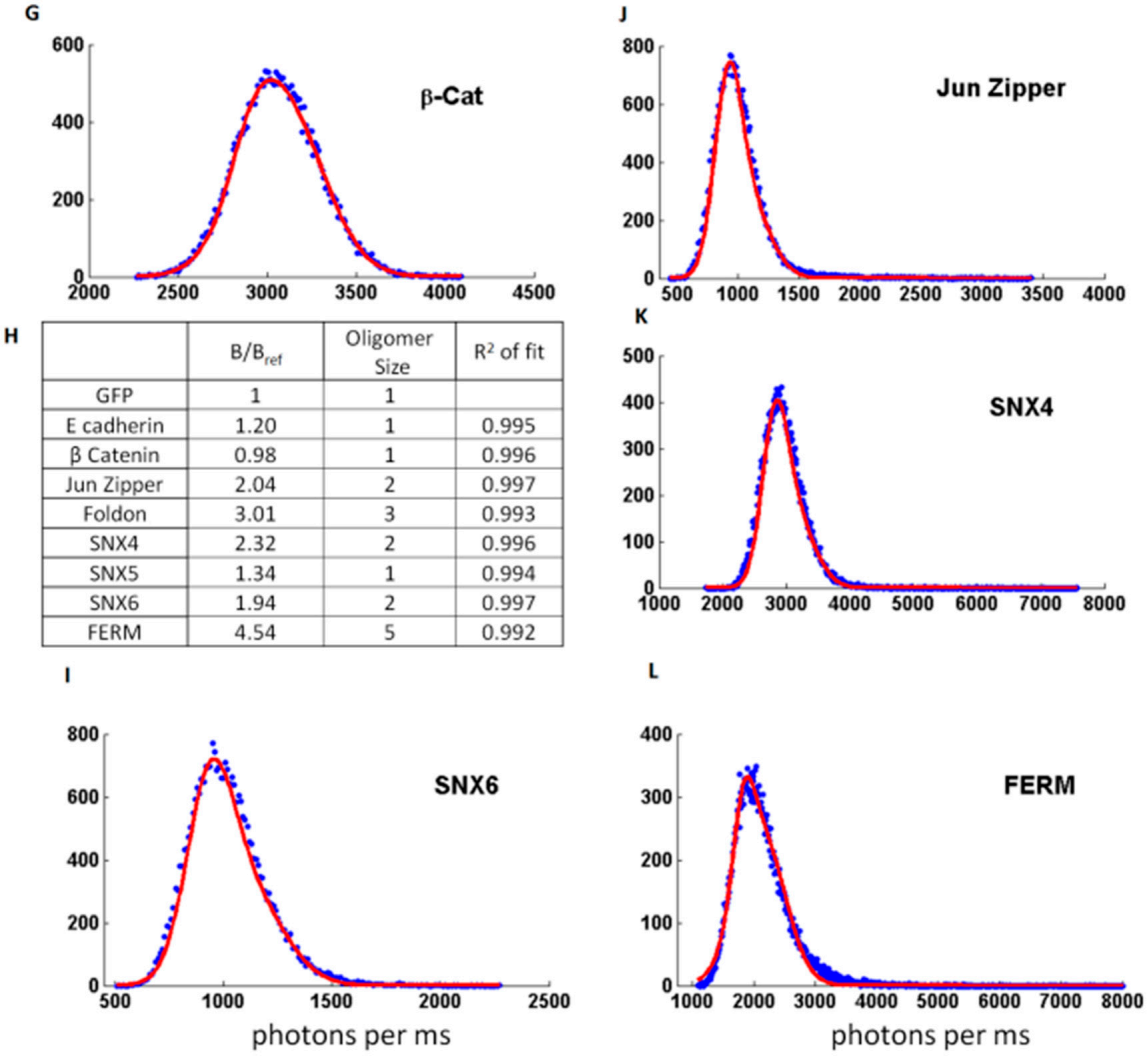
(**A**,**B**) Typical fluorescent time traces used for brightness analysis, obtained for GFP (**A**) and GFP-foldon (**B**). The background fluorescence are similar (~400 photons/ms) but the amplitude of the fluctuations are bigger for GFP-foldon than for GFP; (**C**) Histogram showing the number of events corresponding to different fluorescence intensity values (photons per ms or cpms) measured in the time traces shown in (**A**) and (**B**) for GFP (light grey) and GFP-foldon (black). The distribution for GFP-foldon (black) is larger than GFP (light grey); (**D**–**L**) Example of different distribution of values for different proteins for which the oligomerization status was previously described using other techniques. In blue are the experimental data and red is the calculated Gaussian fit; (**H**) Table presenting the calculated oligomeric status of the protein based on brightness analysis (“B/Bref”) compared to the literature (“oligomer size”) and accuracy of the fit (“R^2^ of fit”).

Typical data are presented in Figure 4. In the case of monomeric eGFP (Figure 4A), the fluctuations of intensities correspond to the entry/exit of a single fluorophore through the focal volume. The formation of small oligomers, such as trimers (using the foldon domain), creates a broader distribution of values: the simultaneous displacement of three fluorophores bound together creates fluctuations of 3-times larger amplitude (Figure 4B). This difference is clearly seen on the graphs plotting the distribution of intensities (Figure 4C). The distribution for the trimeric GFP (GFP-Foldon) is wider than the one for monomeric GFP. From these data, the brightness parameters can be extracted with B_mono_ = 19.4 and B_foldon_ = 53.1, giving *N* = 2.8.

The brightness analysis was then tested on proteins from previous published studies [97,98] (Figure 4D–L). E-cadherin and β-Catenin were previously measured to be monomeric in our cell-free extracts [97]. We also tested three members of the Sortin Nexin (SNX) family of proteins, as we previously determined that SNX5 was monomeric, and SNX4 and SNX6 were dimeric [98]. Finally, we used the transcription factor Jun, which forms a dimer stabilized with a Leucine Zipper, and the FERM (F for 4.1 protein, E for ezrin, R for radixin and M for moesin) domain of the protein Janus Kinase 2 (JAK2) [16]. These different proteins tagged with eGFP were expressed in our cell-free extracts and the fluorescence time traces were acquired for 30 s. The brightness distributions were obtained and fitted by a Gaussian; the B values were also calculated and the number of proteins per oligomer was determined. The predictions of dimer or trimer formation show excellent agreement with our previous observations (Figure 4H) using other techniques including single molecule fluorescence at pM concentrations.

Note that in one case, the FERM domain, the calculated data do not fit properly the tail of the experimental distribution and the interpretation of oligomer size is unreliable (Figure 4L). Indeed, in this sample, we observed the presence of rare large aggregates that cannot be properly quantified by this method.

### 2.3. A High-Throughput Screening Tool

Although B cannot be used to determine the size of rare aggregates, it is still able to report on their presence and can therefore be used to monitor protein aggregation for screening purposes. B is a good screening parameter as it is concentration-independent, reproducible, fast to acquire and unaffected by the protein size.

#### 2.3.1. B Is a Concentration-Independent Parameter for a Wide Range of Concentrations

**Figure 5:**
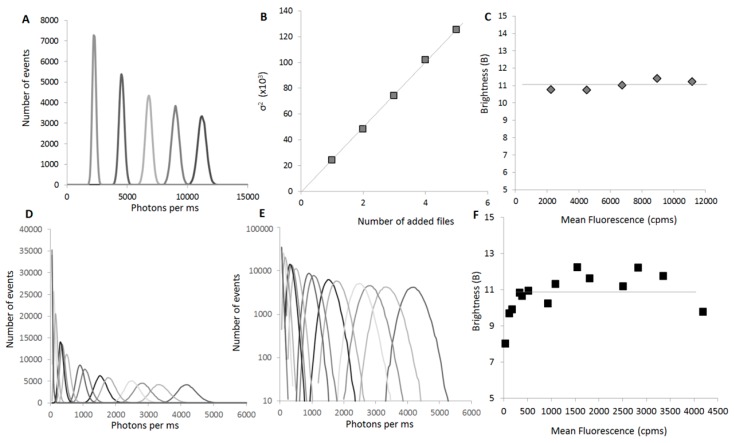
B is independent from the concentration: (**A**) Simulated distributions of values for a serial range of concentrations. To simulate the increase of concentration, we acquired multiple curves for the same sample (GFP monomer). We then artificially added the intensities of 2, 3, 4 or 5 different files, and analyzed the distribution of the sums. As the files are not correlated, we efficiently recreate the increase of concentrations, but not an increase of fluctuations due to oligomer formation. Curves are differentiated by using different shades of grey and black; (**B**) The calculated variance of the distribution (σ^2^) (grey squares) linearly increases with the concentration (or in this case, the number of files used to simulate the concentration increase); (**C**) In this simulation, the brightness parameter (**B**) (grey diamonds) does not vary with the concentration; (**D**) Experimental distributions of values for a serial range of concentrations. Here a GFP-Cherry fusion protein was expressed at different final concentrations and a 60 s fluorescent time trace was acquired for each sample. Curves are differentiated by using different shades of grey and black; (**E**) The distributions of values of (**D**) are presented in a semi-logarithmic scale to show the loss of symmetry of the distributions at low concentrations. Curves are differentiated by using the same shades of grey and black as in (**D**); (**F**) Experimental B values (black squares) as a function of the mean fluorescence. B is indeed independent of the concentration if the mean fluorescence is >400 cpms.

The concentration-independence of B is a built-in property of this parameter, as mentioned previously. To simulate the increase of concentration, multiple curves for the same sample (GFP monomer) were acquired and then the intensities of 2, 3, 4 or 5 different files were artificially added and the distribution of the sums analyzed. As the files are not correlated, we efficiently recreate the increase in concentration, but avoid an increase in the dispersion that would correspond to oligomer formation (Figure 5A–C).

Experimentally, we could determine a range of concentration where the parameter was actually invariant (Figure 5F). This is easily explained when one looks at the profiles of distribution (Figure 5E). At low concentration, the overall distribution, even for a monomer, becomes asymmetrical. This is even more evident when looking at oligomers. For these samples, the passage of large oligomers through the focal volume skews the distribution to higher values, while the lower side of the distribution remains unaffected because the overall protein concentration in the focal volume cannot decrease significantly from the average value.

At high concentrations, the high fluorescence signal can mask small fluctuations and lead to a loss of information. In practice, we found that the B value could be determined in conditions where the fluorescence average signal (µ) was comprised between 400 and 5000 cpms, which in our system corresponds to a range between 4 and 50 nM GFP (Figure 5F).

#### 2.3.2. B Is a Highly Reproducible Parameter

**Figure 6:**
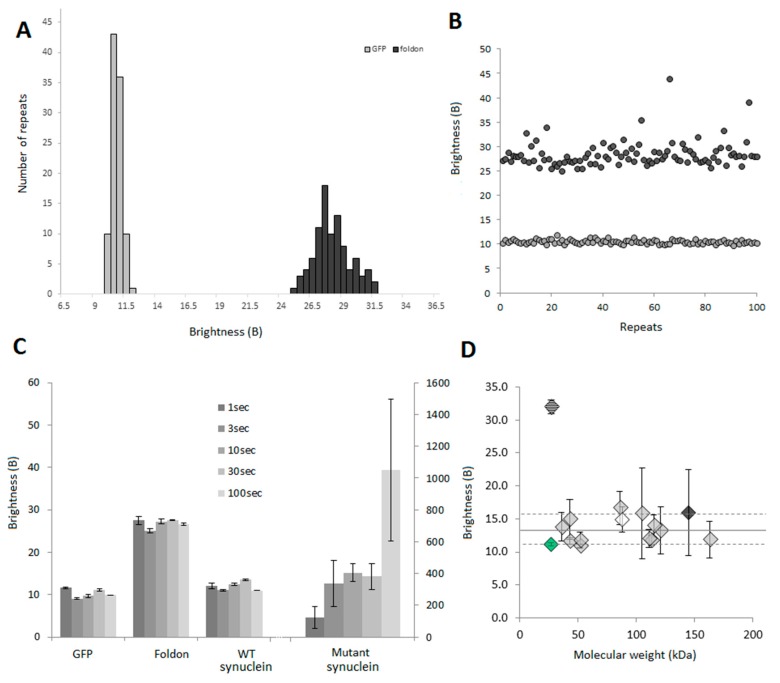
Acquisition of B in confocal spectroscopy: 100 repeats of a 5 s time trace were collected for GFP and GFP-foldon after diluting the proteins to 2000 cpms and B values were calculated. The results are presented in (**A**) in the form of a histogram to show that the distributions of values are narrow, especially for GFP; (**B**) Plotting the B values for the different repeats show the good recurrence of the parameter (grey dots: GFP monomer, black dots: trimeric GFP-foldon); (**C**) B values obtained for different time of acquisition. Time traces of 1 s (10 repeats), 3 s (10 repeats), 10 s (5 repeats), 30 s (5 repeats) or 100 s (3 repeats) were acquired and analyzed. The graph shows average ± SEM. For the synuclein mutant, long traces have to be acquired as the aggregates are relatively rare; (**D**) B as a function of the molecular weight for 16 different proteins (see SI Table S1 for list). Sixteen different proteins were expressed as GFP fusion proteins and 30 s time traces were acquired 3 times for each protein. The graph shows average ± SEM. The dashed diamond corresponds to the GFP foldon, the green diamond is GFP only. The white (Lyn) and dark grey (Gli1) diamonds correspond to proteins that have known interactions with the components of the lysate (lipids and DNA, respectively).

To determine the reproducibility of the brightness analyses, we expressed the GFP monomer and GFP-Foldon and acquired 100 traces of 10 s for each protein. B was calculated for each file and plotted as a distribution or as individual data points to show consistency (Figure 6A,B).

#### 2.3.3. B Can Be Acquired Rapidly

In order to develop a medium-throughput method, acquisition time has to be as low as possible. Different proteins (GFP, GFP-Foldon, WT α-synuclein-GFP, a pathological mutant of α-synuclein E46K-GFP) were expressed in our cell-free system and fluorescent traces were acquired for different amount of time. The B values are presented in Figure 6C. For the calibration, the difference between GFP and GFP-Foldon could be accurately determined even at the smallest acquisition time (1 s). Our monomeric WT α-synuclein behaves similarly to GFP as expected. For highly heterogeneous samples (α-synuclein E46K), longer acquisition times are needed to collect more rare events. However, 10-second long reads are sufficient to reliably establish that a protein is not monomeric provided that aggregation is not an extremely rare event, as would be the case for a prion-like behavior.

#### 2.3.4. B Is Mostly Unaffected by the Size of the Protein

B values were obtained for a panel of 16 N-terminus GFP-tagged proteins with molecular weights ranging from 27 to 163 kDa expected to be monomeric. The experimental B values (Figure 6D) were all found to be between 11 and 16, with an average of 13. These slightly higher values compared to GFP alone (in green), as well as the variability in the data can be explained by the fact that those proteins may not be pure monomers. Indeed, in this panel, proteins that can slightly interact with components in the lysate, lipids (such as Lyn, white diamond) or nucleic acids (Gli1, black diamond) for example, were included.

#### 2.3.5. Oligomers or Aggregates?

**Figure 7:**
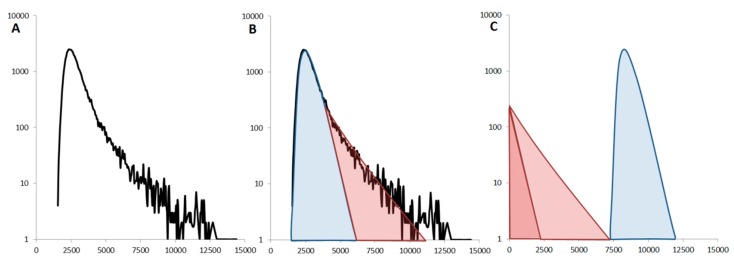
Influence of the large rare aggregates: (**A**) The experimental data obtained for α-synuclein E46K-GFP show an asymmetric distribution of fluorescence values (**B**). The distribution of intensity values can be decomposed into a Gaussian contribution (in blue) and a tail distribution (in red). These two components can then be analyzed separately (**C**). The Gaussian distribution (blue) is analyzed by brightness analysis to give the oligomeric status of the majority of the protein (here monomeric). The tail distribution (red) only contains a small number of events, comparable to data obtained with “pure” (pM) single-molecule acquisition. This can therefore be analyzed using the theory of Photon Counting Histograms (PCH) to give the average number of proteins in the large aggregates.

When a non-monomeric sample is identified, one has to look at the distribution of fluorescent events (and the time traces) to determine if the protein is mainly oligomeric or forms rare large aggregates. If the sample is relatively homogeneous (GFP-Foldon), the distribution is symmetrical. The presence of rare aggregates breaks this symmetry and the distribution of values can be decomposed into two contributions. These include, (1) the fluctuations created by the dominant species, monomer or small, well-defined oligomer, correspond to the symmetrical Gaussian distribution highlighted in blue and (2) the rare events are typically at single-molecule concentrations so their detection is rare and we obtain an exponentially decaying distribution of intensities, as shown in red (Figure 7).

## 3. Examples of Aggregation Assays

### 3.1. Aggregation as a Function of Expression

We use B to determine the aggregation behavior of a protein as a function of the concentration at which it has been expressed.

**Figure 8:**
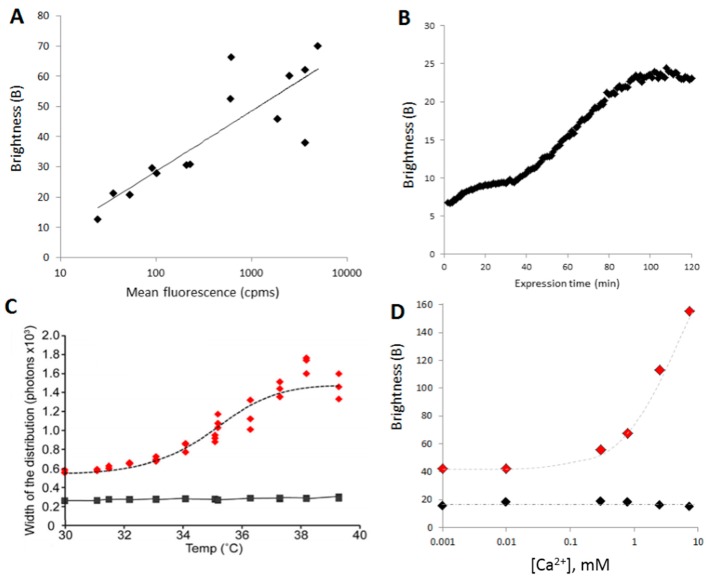
B is a useful parameter in screening assays: (**A**) B as a function of the final concentration of protein. α-Synuclein A30P/A53T, tagged with sGFP at its C-terminus, was expressed at different final concentrations by using a range of dilutions of the template DNA. All expressions were diluted 10 times and 100 s time traces were acquired; (**B**) Variation of B during the expression of the protein. The expression of GFP-foldon was started directly under the microscope by adding the template DNA to the cell-free lysate in the sample holder. Sixty-second time traces were acquired every minute for 2 h; (**C**) B can be used to follow the thermal denaturation of a protein. Here, Munc18-1 WT (black) and mutant (C180Y, red) were expressed as GFP fusion proteins for 2 h and then treated at different temperatures for 1 h. Three time traces of 30 s were acquired for each temperature treatment; (**D**) Changes in aggregation behavior upon calcium treatment. α-synuclein WT (black) and mutant (H50Q, red), tagged with sGFP at the C-terminus, were expressed for 2 h then incubated with different concentrations of Ca^2+^ for 1 h. Three time traces of 30 s were acquired for data point.

To obtain different final concentrations of the proteins (in this case α-synuclein A30P/A53T), a serial dilution of the plasmid encoding the proteins was realized. When expressed in a cell-free expression system, different concentrations of DNA yield different final concentrations of proteins. The samples were then diluted uniformly (11 times in this case) and 45 s time traces were acquired. B could be plotted as a function of the concentration and reveal a “K_d_” of aggregation (Figure 8A).

### 3.2. Time Course of Aggregation

Monitoring aggregation has a function of time, following addition of a reagent. Recording the oligomerization/aggregation status during synthesis of a protein can also be useful as it gives insights into the mechanism of aggregation for example. To monitor aggregation during synthesis, the expression of the GFP-Foldon was initiated by addition of the plasmid to the cell-free extracts. The well was sealed by applying a glass coverslip to limit evaporation, and 60 second-long time traces were obtained every minute for 3 h. Here, B can be plotted as a function of time (Figure 8B).

### 3.3. Determination of Thermal Stability

A very simple application of an aggregation assay is to determine the thermal stability of a protein (also called melting temperature, Tm). Here, upon expression, the cell-free reaction mixes expressing Munc18-1 and one of its mutant [99] were split into 12 samples, which were submitted to a gradient of temperature of (35 ± 5) °C using PCR machine for 30 min. The heated samples were then allowed to cool down to room temperature, diluted and 5 times traces of 10 s were acquired for each sample. The mutant (C118Y) was found to be less stable than WT leading to aggregation of the protein (Figure 8C). As long as the GFP is not thermally denatured (<70 °C), the method is widely applicable.

### 3.4. Small Molecule Inhibitors of Aggregation

A valuable application of an aggregation assay is to screen for drugs that prevent pathological aggregation of proteins. In combination with the cell-free expression system, the assay can be modulated to include the small molecules during protein synthesis (to measure prevention of aggregation) or after expression and aggregation (to find disruptors). Here, we studied the effect of calcium (Ca^2+^) on α-synuclein aggregation, comparing WT and a pathological mutant (H50Q [100]). Titration curves (B *vs.* [Ca^2+^]) were obtained showing that WT was unaffected whereas aggregation of H50Q increased with Ca^2+^ concentration (Figure 8D).

## 4. Discussions

In this “practical guide” to brightness analysis in confocal spectroscopy, we demonstrate that single molecule spectroscopists should take advantage of this simple method to characterize the oligomerization and aggregation propensity of their protein samples. It is often considered that only “true” single measurements performed at extremely low protein concentration can detect sub-populations that would remain hidden in a measurement where molecular properties are averaged. However, as presented above, the brightness method conducted in a “small ensemble” configuration, *i.e.*, at higher protein concentration, gives access to a robust and quantitative measure of protein oligomerization.

In order to go further, it is often important to validate the oligomerization status of the proteins of interest in their cellular context. Indeed, crowding effects, binding partners, post-translational modifications or chaperone activity can modify the oligomerization of proteins in the cell.

As we demonstrated earlier [84], the brightness analysis can be performed easily from cell lysates. In that case, the fluorescent proteins are expressed in different cell lines; the cells are lysed and membrane/nuclei are spun down. The expression levels in the cytoplasm are in general sufficient for a straightforward measurement of brightness on most confocal microscope.

These *in vitro* spectroscopy measurements can be pushed further by turning to cell imaging. Advanced cell microscopy requires more advanced confocal microscope and more expertise from users, but can lead to very valuable information on protein dynamics in time and space. As demonstrated by Enrico Gratton’s laboratory, imaging cells using the Number and Brightness analysis is equivalent to performing many spectroscopic measurements simultaneously, giving access to a pixel-by-pixel map of local concentration and aggregation status of fluorescent protein. In this case, the average pixel intensity is directly linked with the number of fluorophores present, and the variance distinguishes pixels with many monomers from pixels containing a few oligomers.

In cell imaging, the acquisition parameters have to be carefully tuned. For example, the technique has to choose an acquisition rate to capture enough variations of amplitude: if increasing the dwell time increases the apparent brightness, it decreases the amplitude of fluctuations and causes a loss of information. On contrary to diffusing setups, imaging methods have to deal with the existence of immobile molecules or proteins with extremely slow diffusion. To help identify regions containing an immobile fraction, the laser power is varied and the variance measured. For mobile molecules, the variance/intensity increases with intensity, while it remains constant for immobile molecules. The experiments need to take into account a significant background and statistical noise, and the choice of the gain is crucial for proper quantification.

Combined with recent advances in genetically encoded fluorophores and single-molecule imaging techniques, the new developments of Number and Brightness analysis open exciting opportunities to track protein oligomerization in cells with unprecedented temporal and spatial resolution [101,102].

## 5. Conclusions

The brightness parameter has been a valuable tool in single molecule microscopy and can be readily applied to monitor protein aggregation in cells. Here, we described its usefulness when applied to confocal single molecule spectroscopy. We propose to use B as a convenient parameter for the development of aggregation screening assays. Here we also illustrate the use of and changes in the B parameter in understanding various factors affecting protein aggregation. The method takes advantage of genetically-encoded fluorophores to shortcut the necessity of purification/labeling steps. All the examples provided use proteins expressed in a cell-free expression system which enables control of expression levels and reaction time, but samples from other sources can be used, notably lysates from transfected cells.

## Supplementary Materials

Supplementary materials can be found at http://www.mdpi.com/1422-0067/17/5/655/s1.
