# Supplementary material for: Confocal Spectroscopy to Study Dimerization, Oligomerization and Aggregation of Proteins: A Practical Guide
Source: Int J Mol Sci. 2016 Apr 30;17(5):655. doi: 10.3390/ijms17050655 (PMC4881481; doi:10.3390/ijms17050655)
Supplement: Supplementary file 1 [file ijms-17-00655-s001.pdf]

Supplementary Materials: Confocal Spectroscopy to Study Dimerization, Oligomerization and Aggregation of Proteins: A Practical Guide

Yann Gambin, Mark Polinkovsky, Bill Francois, Nichole Giles, Akshay Bhumkar and Emma Sierecki

Table S1. Size and brightness of the proteins expressed in Figure 6D.

Gene Name	Protein Size	Size + GFP	B
CAND1	136	163	11.9
GLI1	118	145	15.9
VAV2	94	121	13.2
STAT3	88	115	13.9
PLCD1	86	113	11.8
HSP90AA	84	111	12.0
MED25	78	105	15.8
HSPD1	61	88	14.9
LYN	59	86	16.6
MED31	16	43	14.9
MED21	16	43	11.7
NEDD8	9	36	13.8
GFP	0	27	11.2
GFP-foldon	0.1	27.1	32.0
GFP-Cm	25	52	10.9
Cm-GFP	25	52	11.8
